# Healing invisible wounds of the Syrian conflict

**DOI:** 10.2471/BLT.16.020116

**Published:** 2016-01-01

## Abstract

Mental health services are becoming more widely available than ever before to the Syrian population in spite of the crisis. Dale Gavlak reports.

Rayyan, a nine-year old Syrian girl, was diagnosed by chance.

“She was hiding behind her depressed and exhausted mother, while I examined her sister who was suffering from malnutrition,” says Dr Waseem Dahkoul, who is working in a community health centre in the Syrian city of Aleppo.

“Rayyan had been suffering with sleep problems and wetting the bed since a missile hit their neighbourhood,” Dahkoul says. “She had no friends and her drawings were full of sadness and images of war.”

“Since she started receiving psychosocial support for symptoms of acute stress, Rayyan has been sleeping better, playing with friends again and doing amazing drawings with roses, trees and butterflies,” he says.

Dahkoul works at one of 130 health centres in 11 of the country’s 14 governorates that are providing mental health services for the many local people in need.

These centres are providing support for about 10 000 people in need every month. Many are suffering deep psychological distress four years into the conflict that has brought widespread displacement, death and destruction and triggered the world's largest humanitarian crisis since the Second World War.

An estimated 13.5 million people need humanitarian assistance, according to the European Commission, and more than 220 000 people have been killed, half of them civilians.

“At least one in every 30 Syrians will be suffering from a severe mental health condition,” says Dr Fahmy Hanna, a technical officer from the World Health Organization’s (WHO) Department of Mental Health and Substance Abuse. 

WHO estimates that, during emergencies, the prevalence of severe mental illnesses, such as psychosis and severe forms of depression increases by 3–4%, and the prevalence of mild to moderate mental disorders, such as depression and anxiety, increases by 15–20%.

Such increases are associated with the psychological pressures brought by the horrors of war, destroyed homes and living in overcrowded conditions. So it is during and after crisis periods that mental health programmes are especially needed.

Before the conflict erupted in 2011, the Syrian mental health-care system was concentrated mainly in three psychiatric hospitals in the country’s two largest cities, Damascus and Aleppo.

About 70 psychiatrists served the entire population of the country, some 23 million people, and a few psychologists offered counselling.

The hospital in Aleppo has since been destroyed, the other two hospitals are inaccessible and most psychiatrists have fled the country.

Over the last two years, WHO has supported the decentralization of the country’s mental health services and helped to rebuild them out of the ruins of the previous centralized model, so that treatment and care is now becoming available at primary care level.

The approach reflects the reform of mental health services all over the world. 

Mental health services in many countries are still confined to large psychiatric hospitals, although some countries have decentralized these services to units attached to general hospitals, as part of sweeping reforms that respect individual rights. 

“It is possible to build mental health services – even in the midst of a crisis,” says Elizabeth Hoff, WHO representative in the Syrian Arab Republic.

Hoff and her team played a key role in supporting Syrian professionals to launch the country’s new decentralized mental health programme.

“A lot of work remains to be done and needs to be funded in the subsequent years,” Hoff adds.

“A lot of work remains to be done and needs to be funded in the subsequent years.”

Elizabeth Hoff

Only 16% (130) of 832 health centres that are still functioning across the divided country have doctors trained to use the WHO Mental Health Global Action Programme Intervention Guide – the mhGAP guide.

These doctors are trained to recognize and diagnose most mental health conditions, including depression, psychotic disorders, substance use disorders and child mental health problems.

Free treatment and care are available at the 130 centres, either provided by the doctors or by psychologists.

For Hoff, a major challenge of the new Syrian programme is to expand mental health services to opposition-controlled areas in the country.

A self-help programme is being devised for people in these hard-to-reach areas.

Hoff attributes the success of the Syrian programme to the fact that it was designed and developed by local health experts trained by WHO in collaboration with all the key health-care providers. 

Usually requests for WHO to help build mental health-care systems that are community-based, integrated into primary health care and responsive to the community’s needs come after an emergency is over, says Dr Hanna. But the story in the Syrian Arab Republic is different.

In late 2013, experts from WHO gathered in Beirut, the capital of Lebanon, to devise a plan to address the growing mental health needs in the country.

Their recommendation – to rebuild decentralized mental health services – was based on WHO’s previous experience and lessons learnt from other emergencies. 

The plan was adopted in agreement with the Syrian Ministry of Health, the United Nations refugee agency (UNHCR), the United Nations Children’s Fund, and nongovernment organizations including the International Medical Corp and the Syrian Arab Red Crescent, 

Hanna, who at the time was working on the mental health situation during Libya’s revolution, and Dr Eyad Yanes were among those representing WHO at the Beirut meeting.

Yanes now works with WHO as National Mental Health Officer, coordinating a network of psychiatrists in the Syrian Arab Republic and helping to train them to provide mental health care and treatment.

First, the mhGAP guide was translated and adapted to the Syrian context. 

Then in 2014 Yanes and his colleagues from WHO helped to train some 570 non-specialist doctors and nurses and now there are about 700 health workers in primary and secondary health-care centres providing mental health care based on WHO’s guidelines.

Yanes and his colleagues also trained health-care providers working for the Red Crescent Society and other nongovernmental organizations, who are working on both sides of the conflict.

“For the first time, trained health-care professionals worked in more than 100 health-care facilities and in places heavily affected by the conflict such as Damascus (including its rural outskirts), Aleppo, Homs, Hama, Latakia, Tartous, Quneitra and Hasakeh,” says Hanna.

In addition, WHO continued on-the-job training for the health-care providers through a team of Syrian national supervisors, who provide technical support by making onsite visits and/or by communicating through social media such as Facebook, Whatsapp and Skype, in areas limited by the security situation.

This team is in turn supported by international supervisors based outside the country, Hanna explains.

The training covers basic and advanced counselling, cognitive behaviour therapy and family therapy in addition to the prescription of medicines for some mental health conditions.

For Syrian gynaecologist Dr Mouna Farhood, the mhGAP guide training has transformed her relationship with patients and with her own family.

“I learned to identify mental health problems in my patients by asking them about their feelings and sleeping habits,” says Farhood.

For Farhood it also takes time to build trust. One of her patients, a 24-year-old undergraduate, told her on her third visit that she had attempted to commit suicide twice with a knife.

“Her life changed after she started treatment on anti-depressants and with psychosocial support. She successfully completed her first year of college and won a scholarship to continue studying dental medicine in Germany,” Farhood says.

A major challenge for the programme is the stigma attached to mental illness.

“Things are changing. I see that my patients accept now that it is OK to be depressed, they do not feel ashamed to seek help,” says Farhood.

Dahkoul echoes her view: “The mhGAP experience turned me into my patients’ friend and opened new horizons professionally.”

A self-help programme is also being launched for people suffering from distress in hard-to-reach areas. The programme is due to be piloted among a small group of physicians and other health workers early this year and depending on the results may be extended.

WHO has also helped to produce a self-help book with accompanying audio materials so that practitioners can train themselves.

Yanes says that before the current crisis, while working for the Syrian health ministry, he proposed establishing psychiatric units in general hospitals without success. “Now during the crisis, we will have four such units next year.”

More training is planned for 2016 for both general practitioners and nurses in order to build and bolster a middle-level cadre of service providers. 

Still, Hanna and others acknowledge that the mental health needs in the country are huge.

“This programme is a good initiative that needs to be sustained and scaled-up.”

Fahmy Hanna

“This programme is a good initiative that needs to be sustained and scaled-up in different Syrian governorates to be able to cover all needs,” Hanna says.

**Figure:**
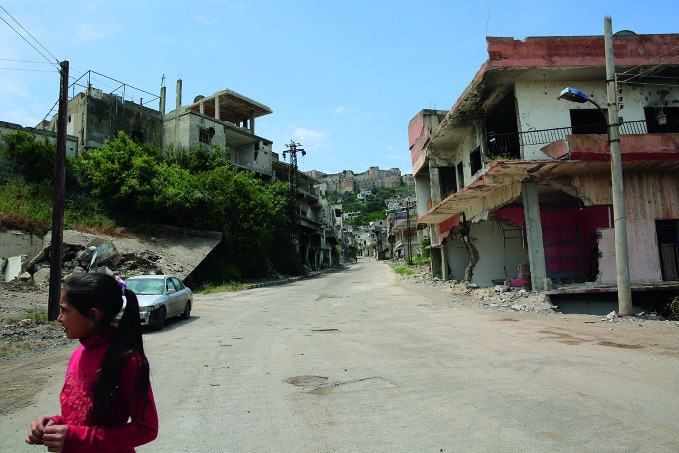
Al Hosn village near the Syrian central city of Homs. A year has passed since the end of the clashes over the village, but only about 1000 people have returned out of more than 40 000 people who used to live there.

UNHCR/Vivian Tou’meh

**Figure:**
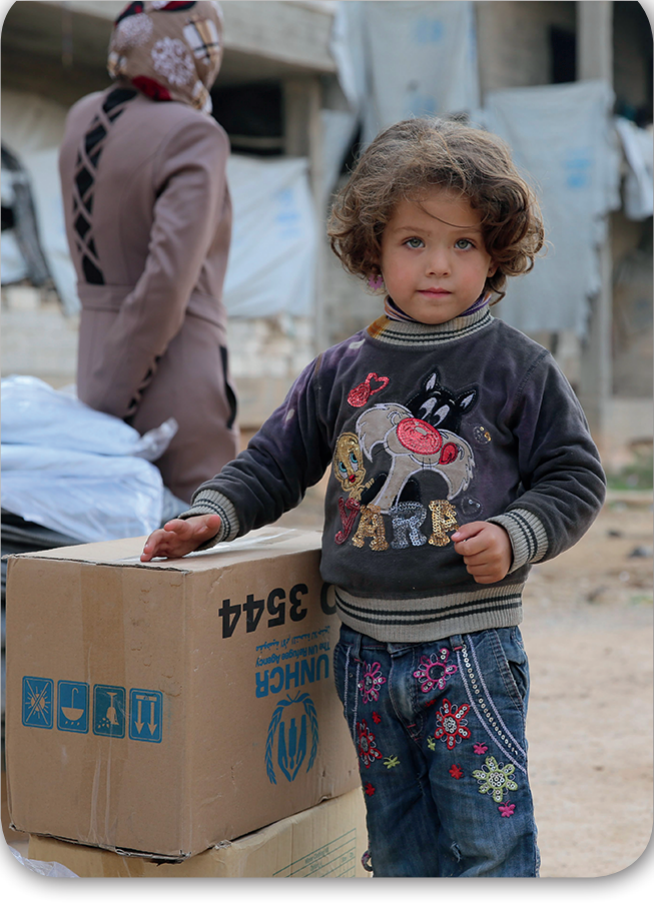
A girl displaced by the fighting now living in Aleppo. She and many others are benefiting from more than 1000 so-called private shelters in the city run by UNHCR, where recreational activities, psychosocial support and primary health care are available.

UNHCR/Bassam Diab

