# Corrigendum

**DOI:** 10.2471/BLT.16.100216

**Published:** 2016-02-01

In Volume 94, Issue 1, January 2016, page 6, the ninth paragraph should read: “WHO estimates that, during emergencies, the prevalence of severe mental illness, such as psychosis and severe forms of depression increases from 2–3% to 3–4%, and the prevalence of mild to moderate mental disorders, such as depression and anxiety, increases from 10% to 15–20%”.

